# Adverse Events in Primary Percutaneous Coronary Angioplasty With Drug-Eluting Stents Compared With Drug-Coated Balloons: A Retrospective Outlook

**DOI:** 10.7759/cureus.8500

**Published:** 2020-06-08

**Authors:** Hamid Sharif Khan, Jahanzeb Malik, Muhammad Mohsin, Asim Javed, Muhammad Umar Farooq

**Affiliations:** 1 Cardiology, Rawalpindi Institute of Cardiology, Rawalpindi, PAK

**Keywords:** stemi, macce, des, dcb

## Abstract

Objective

To compare drug-eluting stents (DES) with drug-coated balloons (DCBs) in terms of major adverse cardiovascular and cerebrovascular events (MACCE) in patients who have undergone primary percutaneous coronary intervention (PPCI).

Methodology

Out of 210 angioplasties in six months, 80 patients were included; 40 in DES and 40 in DCB, respectively. All had a successful PPCI. It was defined as the achievement of thrombolysis in myocardial infarction (TIMI) grade II/III with <20% residual stenosis for the DES and TIMI grade II/III with <30% residual stenosis for the DCB. Any subsequent MACCE during the ensuing six months were assessed from emergency/outpatient records during their subsequent hospital visits, hospital registry, and telephonic interviews.

Results

The mean age in the DES group was 54.83 + 8.72 years while it was 56.8 + 8.9 years in the DCB group. The left anterior descending artery (LAD) was the culprit artery in the majority of the cases in both groups. The mean diameter of DES and DCB was 3.17 + 0.38 mm and 2.75 + 0.53 mm, respectively. Overall, 15 adverse events were seen in the DES group and 16 in the DCB group. The difference was insignificant (P-value = ≥ 0.999). There were nine hospitalizations due to chest pain in the DES group and eight in the DCB group. Total vessel revascularization (TVR) was seen in two patients in the DES group and three patients in the DCB group. None of the patients suffered a stroke. All variables of MACCE were non-significant (P-value = ≥ 0.999).

Conclusion

DCB appears to be non-inferior to DES in PPCI at a median follow-up of six months.

## Introduction

Acute myocardial infarction (AMI) is caused by the occlusion of a coronary artery due to the rupture or erosion of a plaque with thrombus formation [1]. Primary percutaneous coronary intervention (PPCI) is the standard treatment of choice in AMI patients presenting within 12 hours of the onset of chest pain [2]. Drug-eluting stents (DES) are implanted in the blocked arteries after the restoration of thrombolysis in myocardial infarction (TIMI) flow during PPCI [3]. Various large trials have proven DES to reduce repeat revascularization, however, they do not reduce mortality as compared to balloon angioplasty alone and it increases the long-term risk of stent thrombosis and stent restenosis [4-5].

The drug-coated balloon (DCB) has emerged as an alternative treatment option to treat coronary artery stenosis [6]. DCBs are semi-compliant balloons with a chemotherapeutic, anti-proliferative drug (commonly paclitaxel) incorporated with an excipient to facilitate the drug transfer upon inflation of the balloon [7]. The presence of drugs with antiproliferative properties helps reduce the potential risk of an inflammatory response to a metal platform seen in cases of stents, thus reducing the risk of stent restenosis and stent thrombosis [8].

Compared to DES, coronary artery intervention with a DCB is a cumbersome procedure involving proper lesion preparation with pre-dilatation of the vessel with at least equal size as the semi-compliant balloon [9]. Special care is needed in the handling of DCB to avoid the dissipation of the antiproliferative drug before positioning it at the lesion. Prolonged inflation (usually 60 seconds or more) at a nominal pressure is advised to ensure adequate drug delivery at the lesion and ensuring there is no edge dissection, which may require stenting as a bailout if it limits blood flow in the coronary artery [10].

Several studies have demonstrated DCB to infer benefits during elective angioplasties of side branch occlusions [10-11]. Data are limited in patients with AMI. In this study, we sought to demonstrate the major adverse cardiovascular and cerebrovascular events (MACCE) among AMI patients undergoing PPCI with DES and DCB as a comparison in a tertiary cardiac center of Pakistan with a median follow-up of six months.

## Materials and methods

In this retrospective observational study, data collection was started after approval from the research and ethics review committee, Rawalpindi Institute of Cardiology. Records of patients were extracted from the in-house cath-recording system from July 2019 to December 2019, a total period of six months.

All patients were preloaded with aspirin 300 mg and clopidogrel 600 mg. Inclusion criteria were a successful PPCI procedure either with a DES (Xience Expedition, Abbott Laboratories, Abbott Park, Illinois) or a DCB (SeQuent Neo, B. Braun, Melsungen, Germany) at the discretion of the primary operator. Successful PPCI was defined as an achievement of TIMI grade II/III flow with <20% residual stenosis for the DES group and TIMI grade II/III flow with <30% residual stenosis for the DCB group. Both groups were started on aspirin 75 mg once daily and clopidogrel 75 mg once daily. Their risk factors and baseline parameters were noted from their hospital records. Patients who were lost to follow-up or non-compliant to treatment and patients who were deferred stenting were excluded. Patients with comorbidities like diabetes, hypertension, and chronic kidney disease were also excluded.

Any subsequent MACCE including a history of stroke or transient ischemic attack (TIA), death due to AMI, re-infarction, target vessel revascularization (TVR), or hospital admission due to acute coronary syndrome (ACS) during the ensuing six months were assessed from the emergency/outpatient records during their subsequent hospital visits, hospital registry, and telephonic interviews.

Statistical Package for Social Sciences (SPSS) version 26 (IBM Corp., Armonk, NY) was used for data analysis. Continuous variables were expressed as mean ± standard deviation (SD) and categorical variables as a percentage. The chi-square with the Fisher exact test was carried out for a comparison of categorical variables, respectively. P-value of less than 0.05 was considered significant.

## Results

There were a total of 210 angioplasties done in six months. Out of these, 130 patients were excluded based on comorbid conditions, lost to follow-up, or non-compliance to dual antiplatelet therapy (DAPT). Eighty patients were included with 40 who had a DES in place and 40 had DCB angioplasty done. The baseline characteristics and patient demographics are shown in Table 1.

**Table 1:** Demographics and baseline characteristics Drug-eluting stent (DES), drug-coated balloon (DCB), left anterior descending artery (LAD), left circumflex artery (LCX), right coronary artery (RCA), single-vessel coronary artery disease (SVCAD), double-vessel coronary artery disease (DVCAD), triple-vessel coronary artery disease (TVCAD), coronary artery disease (CAD)

Baseline characteristics	DES group	DCB group
Age	54.83 yrs + 8.72	56.8 yrs + 8.9
Gender	Male: 77.5% (n=31) Female: 22.5% (n=09)	Male: 72.5% (n=29) Female: 27.5% (n=11)
Culprit artery	LAD: 52.5% (n=21) RCA: 22.5% (n=09) LCX: 25.0% (n=10)	LAD: 52.5% (n=21) RCA: 25.0% (n=10) LCX: 22.5% (n=09)
Overall CAD	SVCAD: 35.0% (n=14) DVCAD: 52.5% (n=21) TVCAD: 12.5% (n=05)	SVCAD: 27.5% (n=11) DVCAD: 55.0% (n=22) TVCAD: 17.5% (n=07)
Multi-vessel disease: Involvement of more than two coronary arteries	65.0% (n=26)	72.5% (n=29)
Mean stent/balloon diameter	3.17 + 0.38 mm	2.75 + 0.53 mm

 The overall difference in MACCE at six months is tabulated in Table 2.

**Table 2:** Comparison of MACCE in the two subgroups at six months Acute coronary syndrome (ACS), acute myocardial infarction (AMI), target vessel revascularization (TVR), major adverse cardiovascular and cerebrovascular events (MACCE), drug-eluting stent (DES), drug-coated balloon (DCB)

MACCE Component	DES Group	DCB Group	
Death	2.5% (n=01)	5.0 % (n=02)	
Hospitalization due to ACS	22.5% (n=09)	20.0% (n =08)	
Target Vessel Revascularization (TVR)	5.0 % (n=02)	7.5% (n=03)	
AMI (including stent thrombosis)	7.5% (n=03)	7.5% (n=03)	
Overall MACCE	37.5% (n=15)	40.0% (n=16)	

Overall, 15 adverse events were recorded in the DES group and 16 in the DCB group. When the individual MACCE components were compared in the two groups, the difference was found to be clinically insignificant. None of the patients suffered a stroke. Overall, MACCE was insignificant between the two groups.

## Discussion

PPCI with a DES is considered the treatment of choice in patients with AMI [2]. Stenting with a DES reduces target vessel revascularization (TVR) but there is no benefit in aborting recurrent AMI [4]. This was shown in a study that demonstrated an increased incidence of stent thrombosis among AMI patients treated with DES as compared with balloon angioplasty alone, thus paving a potential role of a DCB in PPCI [5]. The presence of a drug on the semi-compliant DCB provides a potential advantage in reducing the potential risk of in-stent restenosis or stent thrombosis [12].

Patients presenting with AMI usually have disease involving more than one coronary artery as demonstrated by a study in which 60% of patients had multivessel disease [13]. Similarly, in our study, about two-thirds of the patients had the involvement of more than one coronary artery, with a prevalence of 65.0% and 72.5% in the DES and DCB groups, respectively.

Several local studies have been conducted over the last few years on DCB but mostly they have been in the form of either a registry or a cohort with a small sample size [14]. Several studies showed the efficacy of DCB in various subsets of coronary artery lesions [15]. But the literature on AMI is limited as far as DCB is concerned.

This was a pilot study done in Pakistan, comparing the short-term outcomes in the form of MACCE among AMI patients treated with DES and DCB. As seen in our study, the overall MACCE at six months was similar in the two groups, with a P-value of > 0.818. Similar results were seen in a study that found PPCI with DCB to be a safe and feasible option and considered non-inferior to DES in the AMI subset of patients [16]. The Drug-Eluting Baloon In Acute Myocardial Infarction (DEB-AMI) trial found no difference in MACCE at six months among patients treated with DCB only PPCI when compared with bare-metal stent (BMS) only PPCI [17]. The main reason for the similar MACCE was the timely management of the patients and the ability to achieve TIMI III blood flow in both the subgroups.

In our study, individual components of MACCE were not significantly different in the two groups. Death was seen in 5.0% and 2.5% of the DCB and DES groups, respectively. Similarly, the incidence of re-infarct at six months was homogeneous in the two groups, at 7.5% each. Related results were seen in a trial that showed a 30-day mortality of 2.4% in the DCB subgroup [18].

The incidence of TVR was seen as 7.5% in the DCB group and 5.0% in the DES group. The difference was, however, not significant. In one study, TVR at 90 days was found to be 3.3% with a DCB [19]. It is lower as compared to our study because we calculated the risk at six months. Being a non-randomized, single-center study, it has the limitations of small sample size and median time frame to follow-up. Further multi-centered randomized control trials are needed to assess the efficacy of DCB in patients of AMI.

## Conclusions

DCB appears to be non-inferior to DES in AMI patients undergoing PPCI at six months. The difference in MACCE between the two groups is insignificant in terms of death, target vessel revascularization, stroke, reinfarction, and hospitalization. DCB appears to be safe at a median follow-up of six months.
